# Modulation of lung CD11b^+^ dendritic cells by acupuncture alleviates Th2 airway inflammation in allergic asthma

**DOI:** 10.1186/s13020-025-01119-9

**Published:** 2025-05-22

**Authors:** Mi Cheng, Pan-Pan Shang, Dan-Dan Wei, Jie Long, Xue Zhang, Quan-Long Wu, Gabriel Shimizu Bassi, Yu Wang, Yan-Jiao Chen, Lei-Miao Yin, Yong-Qing Yang, Yu-Dong Xu

**Affiliations:** 1 Shanghai Research Institute of Acupuncture and Meridian,Yueyang Hospital of Integrated Traditional Chinese and Western Medicine, Shanghai University of Traditional Chinese Medicine, Shanghai, China; 2 School of Rehabilitation Science, Shanghai University of Traditional Chinese Medicine, Shanghai, China

**Keywords:** Allergic asthma, Acupuncture, Th2 cell, Dendritic cells, Type 2 innate lymphoid cells, Airway epithelium, Alarmin cytokines, Immunoregulation

## Abstract

**Background:**

Allergic asthma features Th2-mediated airway inflammation, with dendritic cells (DCs) playing a crucial role. Acupuncture shows promise in modulating immune responses, yet its mechanisms in allergic asthma are not well understood. This study explores how acupuncture alleviates Th2 airway inflammation by modulating lung CD11b^+^ DCs.

**Methods:**

Female BALB/c mice were assigned to control and asthma model groups, with subsets receiving acupuncture at *Dazhui* (GV14), *Fengmen* (BL12), and *Feishu* (BL13). We evaluated airway hyperresponsiveness (AHR), immune cell infiltration, histological changes, Th2 cytokine production, and serum IgE levels. The effects of acupuncture serum on T cell activation and bone marrow-derived dendritic cells (BMDCs) were assessed. The role of CD11b^+^ DCs was analyzed using flow cytometry, cytokine assays, and adoptive transfer experiments. Epithelial-derived alarmins and type 2 innate lymphoid cells (ILC2s) were also examined.

**Results:**

Acupuncture significantly reduced AHR, immune cell infiltration, goblet cell hyperplasia, and serum IgE levels in HDM-induced allergic asthma. It also suppressed Th2 cytokine production and differentiation. While acupuncture serum did not directly affect T cell activation, it modulated BMDC activity. Adoptive transfer of acupuncture-treated lung DCs into asthmatic mice reduced Th2 cell recruitment and ameliorated airway inflammation. Acupuncture reduced the population of lung CD11b^+^ DCs and downregulated the expression of activation markers such as CD86 and OX40L, along with Th2-promoting chemokines CCL17 and CCL22. Furthermore, it influenced CD11b^+^ DC migration by modulating CCR7, CCL2, and CCL8 expression. Acupuncture suppressed epithelial-derived alarmins IL-25, IL-33, and TSLP, attenuating ILC2 accumulation and activation, which indirectly affected CD11b^+^ DCs and Th2 responses.

**Conclusions:**

Acupuncture alleviates Th2 airway inflammation in allergic asthma by modulating lung CD11b^+^ DC activities. These findings provide new insights into acupuncture-based therapeutic strategies for asthma.

**Supplementary Information:**

The online version contains supplementary material available at 10.1186/s13020-025-01119-9.

## Introduction

Allergic asthma is a chronic inflammatory disease characterized by airway hyperresponsiveness (AHR), mucus overproduction, and immune cell infiltration, particularly by T helper 2 (Th2) cells. Th2 cells release cytokines such as interleukin (IL)−4, IL-5, and IL-13, which drive eosinophilic inflammation and play a central role in asthma pathophysiology [1]. Current asthma treatments primarily focus on suppressing Th2-mediated inflammation but often face limitations, including drug resistance and inadequate symptom control, especially in severe cases [2]. A critical drawback of these treatments is their limited impact on upstream processes of Th2 immune responses, particularly the differentiation and activation of dendritic cells (DCs), which are essential for antigen presentation.

DCs, particularly CD11b^+^ DCs, are pivotal in initiating and propagating Th2-mediated immune responses during allergic inflammation. These cells capture allergens and present them to T cells while producing key cytokines that drive the differentiation of naïve T cells into Th2 cells, thereby exacerbating airway inflammation [3, 4]. Group 2 innate lymphoid cells (ILC2s) also significantly contribute to shaping the immune environment of the airways by responding to epithelial cell-derived alarmins such as IL-25, IL-33, and thymic stromal lymphopoietin (TSLP) [5]. Activated ILC2s produce cytokines that further stimulate CD11b^+^ DCs, leading to an amplification of Th2 airway inflammation [6]. Therefore, targeting the interaction of CD11b^+^ DCs and ILC2s, as well as their associated cytokines, represents a promising therapeutic strategy for asthma management.

Acupuncture, a non-invasive neural stimulation technique, has gained attention for its immunomodulatory properties presenting and potential therapeutic benefits in managing inflammatory conditions such as asthma [7]. Among the acupoints commonly used for asthma treatment, *Dazhui* (GV14), *Fengmen* (BL12), and *Feishu* (BL13) have been prioritized due to their demonstrated effectiveness in improving respiratory function [8]. Clinical evidence and experimental studies indicate that acupuncture at these acupoints alleviates allergic asthma symptoms by modulating the Th2 inflammatory response [9–11]. However, the mechanisms by which acupuncture regulates lung DC and ILC2 functions in allergic asthma remain incompletely understood.

This study investigates the role of acupuncture in modulating CD11b⁺ DC populations and functions in allergic asthma, hypothesizing that acupuncture attenuates Th2 airway inflammation by limiting CD11b⁺ DC-mediated immune activation and subsequent Th2 cell recruitment. Additionally, the study explores whether acupuncture indirectly influences CD11b⁺ DCs by regulating ILC2 activity. The findings are expected to provide mechanistic insights into acupuncture’s immunomodulatory effects in asthma, supporting the development of novel therapeutic approaches for airway diseases.

## Methods

### Mice

Female BALB/c mice (6–8 weeks old) were purchased from Beijing Vital River Laboratory Animal Technologies Co., Ltd and randomly assigned to four groups: normal control (NC), asthma model (AS), asthma model with acupuncture treatment (AA), and normal control with acupuncture treatment (NA). Mice were housed in a specific pathogen-free facility under a 12 h light/dark cycle, at room temperature with 55 ± 5% humidity, in ventilated cages with ad libitum access to food and water. All procedures were approved by the Institutional Animal Care and Use Committee of Shanghai University of Traditional Chinese Medicine (approval no. PZSHUTCM211101012) and followed the Guide for the Care and Use of Laboratory Animals.

### Asthma model

Mice received anesthesia with 2% isoflurane and intranasal sensitization with 10 μg of house dust mite (HDM) extract (Greer Laboratories, Lenoir, NC) on days 0 and 7, followed by daily 20 μg HDM challenges on days 14–18. Control mice received saline in equivalent volumes. Mice were euthanized 48 h after the final HDM exposure for analysis.

### Acupuncture treatment protocol

**Fig. 1:**
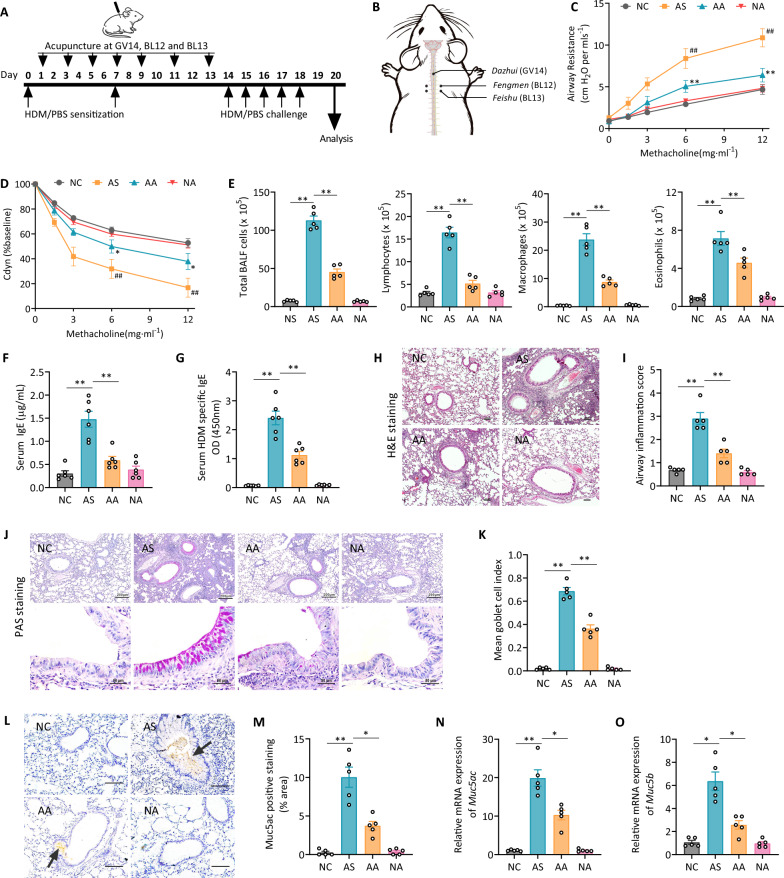
Acupuncture alleviates airway inflammation and hyperresponsiveness in an HDM-induced allergic asthma model. **A** Schematic representation of the experimental timeline for HDM/PBS sensitization and challenge, with acupuncture treatment administered at *Dazhui* (GV14), *Fengmen* (BL12), and *Feishu* (BL13) on designated days. **B** Illustration of acupoint locations used in the study. GV14 is located in the depression below the spinous process of the C7 vertebra, BL12 is positioned in the foveola laterally between the T2 and T3 vertebrae, and BL13 is found in the foveola laterally between the T3 and T4 vertebrae. **C** Airway resistance measured in response to increasing doses of methacholine (normalized to baseline). **D** Dynamic lung compliance assessed in response to increasing methacholine doses (normalized to baseline). **E** Total and differential counts of inflammatory cells (lymphocytes, macrophages, eosinophils) in BALF. **F** ELISA analysis of serum IgE levels, including (**G**) HDM-specific IgE. **H** Representative H&E-stained lung sections depicting inflammatory changes across different groups. Scale bar = 200 μm. **I** Quantification of airway inflammation scores based on H&E staining. **J** PAS staining of lung sections to evaluate goblet cell hyperplasia. Upper scale bar = 200 μm; lower scale bar = 50 μm. **K** Quantification of the mean goblet cell index from PAS staining. **L** Representative images of immunohistochemical staining for Muc5ac in lung tissues, highlighting mucus overproduction. Scale bar = 100 μm. **M** Semi‑quantitative assessment of Muc5ac protein expression in lung tissues. **N**–**O** Relative mRNA expression levels of *Muc5ac* (**N**) and *Muc5b* (**O**) gene in lung tissues. NC: normal control, AS: asthma model, AA: asthma + acupuncture, NA: normal + acupuncture. Data are presented as means ± SEM; n = 5–6 mice/group; *P < 0.05, **P < 0.01 as indicated. For C and D, ##*P* < 0.05, ##*P* < 0.01 compared with NC; *P < 0.05, **P < 0.01 compared with AS

Following anesthesia with 2% isoflurane, mice in the AA and NA groups received manual acupuncture beginning after sensitization and continuing every other day for two weeks (Fig. 1A). Mice in the NC and AS groups underwent identical isoflurane anesthesia procedures but did not receive acupuncture treatments. Acupoints GV14, BL12, and BL13 were chosen based on traditional Chinese medicine principles for asthma treatment. Mouse acupoint locations were determined according to WHO guidelines for human acupoints (Fig. 1B). The acupuncture procedure was performed following the standardized methodology established in our previous studies [12, 13]. Disposable stainless steel needles (13 mm, 0.30 mm diameter; Suzhou Medical Appliance Factory, China) were inserted approximately 2.5–3 mm deep and rotated 360º back and forth at 200 rotations per minute for 30 s, manipulated every 3 min, and withdrawn after 30 min. All treatments were performed by licensed, experienced acupuncturists specifically trained in the acupuncture protocol and were blinded to group assignments.

### Airway hyperresponsiveness measurement

Dynamic lung resistance (R_L_) and compliance (C_dyn_) were measured 48 h after the final HDM challenge using the Buxco FinePointe RC system (DSI, St. Paul, MN, USA). Mice were anesthetized with 1% pentobarbital sodium (10 mL/kg), tracheotomized, and intubated with an 18-gauge catheter attached to a ventilator set to 140 breaths/min, a tidal volume of 0.2 mL, and a positive-end expiratory pressure of 2 cm H_2_O. After a 5 min stabilization, baseline readings were taken with aerosolized PBS, followed by exposure to methacholine (MCh) at increasing concentrations (1.5, 3, 6, and 12 mg/mL; Sigma-Aldrich). R_L_ and C_dyn_ values were recorded for 3 min after each MCh challenge and expressed as changes from PBS baseline.

### Blood samples collection

Anesthetized mice underwent cardiac puncture for blood collection. Samples were allowed to clot at 4 °C for 3 h, followed by centrifugation at 3,000 rpm for 10 min to obtain serum. Part of the serum was used for IgE level detection, while the remaining serum was heat-inactivated at 56 °C for 30 min, filtered through a 0.2 μm syringe filter for sterilization, and used in subsequent cellular experiments. For cellular assays, serum was pooled from a representative subset of mice in each group to reflect the average treatment response and minimize inter-individual variability, ensuring experimental consistency.

### Bronchoalveolar lavage fluid (BALF) analysis

Intubated mouse tracheas were lavaged three times with 1 mL warm PBS to obtain BALF from the right lung. The fluid was centrifuged at 500 g for 10 min at 4 °C. Cell-free supernatants were used for cytokine and chemokine quantification via ELISA kits, while cell pellets were resuspended in 0.1 mL PBS with 1% FBS for leukocyte differential counts using a BC-5000 Vet auto hematology analyzer (MINDRAY, Shenzhen, China).

### Lung histology

Lungs were fixed in 10% formalin for 24 h, dehydrated, embedded in paraffin, and sectioned at 4 µm using a Leica microtome (Leica, Germany). Sections were dewaxed and stained with hematoxylin and eosin (H&E) or periodic acid Schiff (PAS). Inflammatory cell infiltration, tissue damage, goblet cell metaplasia, and mucus production were quantified using a Nikon 80i microscope, following established protocols [14]. Lung inflammation was scored semi-quantitatively (0–4), with scores representing increasing peribronchial/perivascular inflammatory cell layer thickness. Goblet cell hyperplasia was quantified by counting PAS-positive cells, expressed as a percentage of total epithelial cells. Ten fields per section were scored blindly by two pathologists.

### Immunohistochemistry for Muc5ac in lung tissue

Lung sections were deparaffinized, rehydrated, and underwent antigen retrieval in citrate buffer (10 mM, pH 6.0) at 95 °C for 20 min. After cooling, sections were washed with PBS and treated with 3% hydrogen peroxide for 10 min to block endogenous peroxidase, followed by 5% normal goat serum to block non-specific binding. Sections were incubated overnight at 4 °C with rabbit anti-Muc5ac antibody (Cell Signaling Technology, Cat: 36623) in PBS with 1% BSA. Following washes, sections were treated with an HRP-conjugated secondary antibody for 30 min and visualized using DAB substrate (Beyotime, Cat: P0203). Stained areas were quantified using ImageJ software to calculate the percentage of Muc5ac-positive staining as an indicator of protein expression.

### Preparation of mouse lung homogenates and single-cell suspensions

After euthanizing mice, lungs were harvested and rinsed in ice-cold PBS. For protein lysates, lung tissue was homogenized in RIPA buffer (Beyotime, Cat: P0013) with protease inhibitors (Roche, Cat: 04693124001) using a tissue homogenizer. Homogenates were centrifuged at 12,000 g for 15 min at 4 °C, and the supernatants were stored at −80 °C. For single-cell suspensions, lung tissue was minced, digested in 1 mg/mL collagenase I (Sigma-Aldrich, Cat: V900891) and 200 U/mL DNase I (Sigma-Aldrich, Cat: 10104159001) in RPMI-1640 medium for 45 min at 37 °C with gentle shaking. The digested tissue was filtered through a 70 µm cell strainer, washed with PBS, and centrifuged at 400 g for 5 min at 4 °C. Red blood cells were lysed with RBC lysis buffer, and cells were resuspended in RPMI-1640 for downstream applications.

### Adoptive transfer of DCs

Lung DCs were isolated via CD11c-positive selection with UltraPure MicroBeads (Miltenyi Biotec, Cat: 130-125-835) as per the manufacturer’s protocol, achieving > 95% CD11c^+^ purity. A total of 1 × 10^6^ DCs from each experimental group were resuspended in 100 μL PBS and adoptively transferred to female, 6–8 week-old naïve BALB/c recipient mice via tail vein injection. Seven days post-transfer, recipients received a 5 day course of 20 µg/mL intranasal HDM, allowing the transferred DCs to encounter antigen within the lung environment. Mice were sacrificed 24 h after the final challenge for further analysis.

### Quantification of HDM-specific IgE

Serum HDM-specific IgE was quantified by using an ELISA kit (BioLegend, Cat: 432404) with modifications. Plates were coated overnight at 4 ℃ with capture antibodies, blocked, and incubated with serum samples for 2 h. Biotinylated HDM extract (CITEQ, Cat: 02.01.88) was added for 1 h, followed by streptavidin-HRP. TMB substrate was used for color development, stopped with 2 M H_2_SO_4_, and plates were read at 450 nm (BioTek, Synergy H1). Optical density values quantified HDM-specific IgE levels.

### ELISA

ELISA kits from BioLegend were used to measure IL-4, IL-5, IL-6, IL-1β, TNFα, TSLP, and IL-25 levels, while Invitrogen kits were used for IL-13 and IL-33. CCL17 and CCL22 levels were quantified using kits from R&D Systems and PeproTech, respectively, following each manufacturer's protocol. The lower and upper limits of detection (LLOD and ULOD) for each ELISA kit are specified in Supplementary Table S1.

### Ex vivo mediastinal lymph node (MLN) cell culture

Single-cell suspensions from MLNs were cultured at 1 × 10^6^ cells/mL in round bottom 96-well plates and exposed to 10 μg/mL HDM for 72 h in RPMI-1640 medium with 10% FBS at 37 °C. Cytokine levels in the culture supernatants were determined via ELISA, and mRNA levels of *Gata3* and *Il4* in MLN cells were analyzed by qPCR.

### Isolation and stimulation of T lymphocytes

After harvesting and mechanically disrupting spleens, single-cell suspensions underwent red blood cell lysis. T lymphocytes were isolated using a negative selection kit (Miltenyi Biotec, Cat: 130-095-130) and cultured in RPMI-1640 with 10% FBS. For activation, T cells were stimulated with 10 μg/mL plate-bound anti-CD3e and 2 μg/mL soluble anti-CD28 mAb (BD Biosciences, Cat: 553294). Flow cytometry assessed T cell activation by measuring CD25 and CD69 surface expression.

### CFSE staining

Isolated T cells were labeled with 0.5 µM CFSE and plated at 1 × 10^6^ cells/well. Cells were pretreated with 10%, 1%, or 0.1% serum from mice or 100 nM cyclosporin A (CsA, Sigma-Aldrich, Cat: SML1018) for 6 h, followed by stimulation with 10 μg/mL anti-CD3e and 2 μg/mL anti-CD28 mAb. Control cells received no treatment. After 72 h at 37 °C in 5% CO₂, flow cytometry analyzed CFSE dilution to quantify T cell proliferation, with lower CFSE intensity indicating higher cell division rates.

### In vitro Th2 cell polarization assay

Naïve CD4^+^ T cells were isolated from splenocytes using a Naïve CD4^+^ T Cell Isolation Kit (Miltenyi Biotec, Cat: 130-104-453), resuspended in RPMI-1640 with 10% FBS, 100 U/mL penicillin, 100 µg/mL streptomycin, and 50 µM β-mercaptoethanol, and seeded at 1 × 10⁶ cells/mL. For Th2 differentiation, cells were activated with anti-CD3e, anti-CD28, 5 ng/mL IL-2, 10 ng/mL IL-4, and 10 µg/mL anti-IFN-γ. Cultures were incubated for 3 days with serum from treated/untreated mice or CsA as a control. Th2-specific markers GATA-3 and IL-4 were measured by qPCR.

### Generation of bone marrow‐derived dendritic cells (BMDCs)

Femurs and tibias were harvested from euthanized mice and sterilized with 75% ethanol. Bone marrow cells were flushed with pre-cooled RPMI-1640 medium, followed by erythrocyte lysis. Cells were resuspended in RPMI-1640 with 10% FBS, 100 U/mL penicillin, and 100 µg/mL streptomycin at 1 × 10⁶ cells/mL and cultured with 20 ng/mL GM-CSF (R&D Systems, Cat: 415-ML) and 10 ng/mL IL-4 (R&D Systems, Cat: 404-ML) at 37 °C in 5% CO₂. On day 3, non-adherent cells and 75% of the medium were replaced. On day 5, half the medium was refreshed. On day 7, non-adherent cells were collected as immature BMDCs or further cultured with LPS to induce maturation.

### Cell uptake assay

BMDCs (1 × 10⁶ cells/mL) were seeded in 12-well plates and treated with 10% mouse serum or 10 µM dexamethasone (Sigma-Aldrich, Cat: D4902) for 6 h. Phagocytic activity was assessed by incubating with 1 mg/mL FITC-dextran (40 kDa, Sigma-Aldrich, Cat: 46945) for 1 h at 37 °C, followed by washing with ice-cold PBS and flow cytometry analysis of FITC fluorescence.

### mRNA analysis

Total RNA from lung or MLN cells was extracted using TRIzol (Invitrogen, Cat: 15596026), reverse-transcribed with RevertAid cDNA Synthesis Kit (Thermo Scientific, Cat: K1622), and analyzed by qPCR using SYBR Green (TOYOBO, Cat: QPK-201) on a LightCycler 96 System. Gene expression was normalized to *Gapdh* using the 2^*−ΔΔCt*^ method. Primer sequences are listed in Supplementary Table S2.

### Flow cytometry analysis

Single cells were blocked with anti-CD16/32 (BD Biosciences, Cat: 553141) in PBS + 2% FBS, then stained with target antibodies for 30 min at 4 °C. Fixable Viability Dye eFluor 780 (Invitrogen, Cat: 65–0865-18) excluded dead cells. Intracellular staining used the Cytofix/Cytoperm Kit (BD Biosciences, Cat: 554714). Flow cytometry was conducted on an Attune NxT (ThermoFisher, MA, USA), analyzed with FlowJo. Antibody details are in Supplementary Table S3.

### Statistical analyses

Data were presented as means ± SEM. Group differences were evaluated using one-way ANOVA, followed by LSD or Games-Howell post-hoc tests, depending on data distribution. For non-parametric data, the Kruskal–Wallis test was applied, with post-hoc comparisons made using Dunnett’s test. While, two-way ANOVA was used to analyze AHR experiments. The statistical significance was set at *P* < 0.05.

## Results

### Acupuncture treatment alleviates HDM-induced allergic asthma

To investigate acupuncture’s impact on HDM-induced allergic asthma, seven acupuncture treatments were administered every other day at GV14, BL12, and BL13 following initial HDM sensitization (Fig. 1A, B). The AS group showed an increased airway R_L_ in response to MCh, whereas acupuncture treatment significantly reduced R_L_, indicating improved pulmonary function (Fig. 1C). Furthermore, C_dyn_, which was markedly decreased in the AS group, was partially restored by acupuncture treatment (Fig. 1D).

Enhanced immune cell infiltration in BALF indicated a strong inflammatory response in the AS group, with significantly elevated numbers of total immune cells, lymphocytes, macrophages, and eosinophils (Fig. 1E). Acupuncture effectively decreased these immune cell counts, indicating its role in mitigating airway inflammation. The AS group exhibited increased levels of total and HDM-specific serum IgE levels, reflecting an enhanced allergic response. However, these levels were significantly lower in the AA group (Fig. 1F, G). Additionally, the spleen-to-body weight ratio, a marker of systemic immune activation, was elevated in the AS group but significantly reduced by acupuncture (Fig. S1 A, B).

Histological analysis confirmed a reduction in peribronchial and perivascular inflammation in the AA group (Fig. 1H, I). PAS staining further revealed that acupuncture alleviated goblet cell hyperplasia and mucus overproduction treatment (Fig. 1J, K). Immunohistochemical analysis of Muc5ac protein (Fig. 1L, M) showed excessive mucus production in HDM-challenged mice, which was significantly reduced in the AA group. This was further supported by decreased *Muc5ac* and *Muc5b* mRNA expression (Fig. 1N, O). No differences were observed between the NC and NA groups across these tests, underscoring acupuncture's specific effects in asthma.

### Acupuncture suppresses Th2 inflammatory response in asthmatic mice

**Fig. 2:**
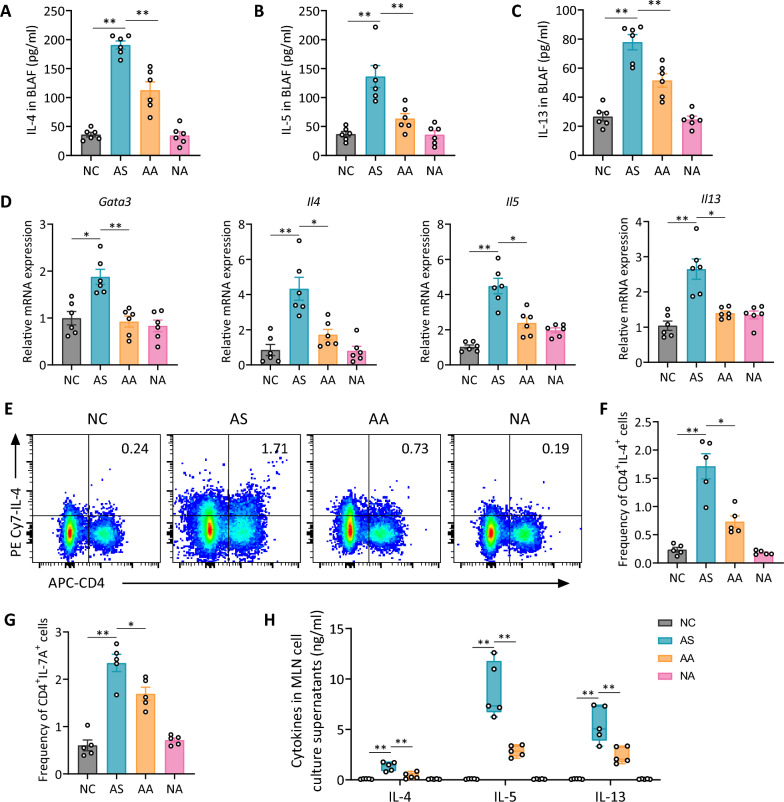
Acupuncture reduces Th2 cytokine production and Th2 cell differentiation in an HDM-induced asthma model. **A**–**C** ELISA analysis of Th2 cytokines IL-4 (**A**), IL-5 (**B**), and IL-13 (**C**) in BALF. **D** Relative mRNA expression levels of *Gata3*, *Il4*, *Il5*, and *Il13* in lung tissues. **E** Flow cytometry analysis of IL-4^+^CD4^+^ T cells (Th2 cells) in lung tissues. **F** Quantification of the percentage of Th2 cells in lung tissues. **G** Percentage of IL-17 A^+^CD4^+^ T cells (Th17 cells) determined by flow cytometry. **H** Cytokine concentrations of IL-4, IL-5, and IL-13 in the supernatants of MLN cell cultures restimulated with HDM in vitro. Data are shown as means ± SEM; n = 5–6 mice/group; *P < 0.05, **P < 0.01 as indicated

To examine the effects of acupuncture on Th2 cell-dominant airway inflammation, we analyzed Th2-related cytokines and transcription factors in HDM-induced asthmatic mice. Acupuncture significantly reduced IL-4, IL-5, and IL-13 levels in BALF compared to the AS group (Fig. 2A–C). Consistently, *Gata3*, *Il4*, *Il5*, and *Il13* mRNA levels were also lower in lung tissue of acupuncture-treated mice, further supporting its inhibitory effect on Th2 activity (Fig. 2D). Flow cytometry analysis showed a marked reduction in CD4^+^IL-4^+^ T cells and CD4^+^IL-17 A^+^ T cells in the lungs of the AA group, indicating that acupuncture modulates both Th2 and Th17 responses in the airway (Fig. 2E–G, Fig. S2B); gating strategy was shown in Fig. S2 A. In addition, MLN cell culture analysis revealed reduced IL-4, IL-5, and IL-13 production following HDM restimulation in the AA group compared to the AS group (Fig. 2H). Overall, these results indicate that acupuncture alleviates Th2 airway inflammation by inhibiting Th2 differentiation and activation.

### Acupuncture serum exhibits no direct effect on T cells but modulates BMDC activity

**Fig. 3:**
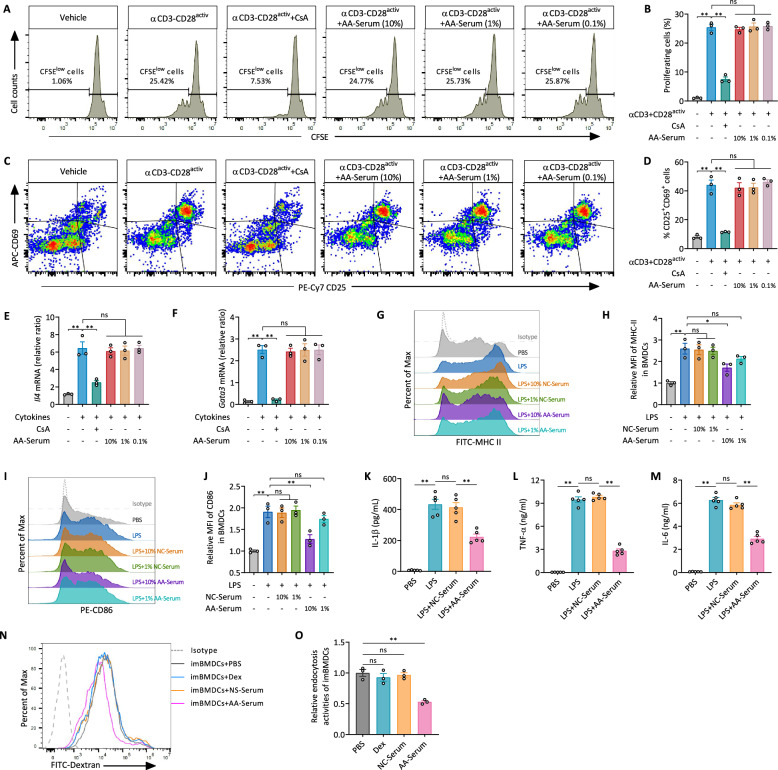
Acupuncture serum does not directly affect T cells but modulates BMDC activity. **A** Flow cytometry analysis of T cell proliferation in response to anti-CD3/CD28 antibodies (αCD3-CD28) stimulation with cyclosporine A (CsA) or varying acupuncture-treated asthma (AA)-Serum concentrations. CFSE dilution reflects the percentage of proliferating cells. **B** Quantification of proliferative cells, showing that AA serum did not significantly suppress T cell proliferation compared to controls. **C** Flow cytometry plots illustrating CD25 and CD69 expression in treated T cells, and **D** quantification of CD25^+^CD69^+^ activated T cells. **E**, **F** Relative mRNA expression levels of *Il4* and *Gata3* in T cells under Th2-polarizing conditions, measured by qRT-PCR. **G** Flow cytometry histograms illustrating MHC II expression in LPS-stimulated BMDCs with normal control (NC)-Serum or AA-Serum, and **H** corresponding relative MFI quantification. **I** Flow cytometry histograms illustrating CD86 expression in LPS-stimulated BMDCs, and **J** corresponding relative MFI quantification. **K**–**M** Levels of IL-1β, TNF-α, and IL-6 in culture supernatants of LPS-stimulated BMDCs treated with AA-serum, assessed by ELISA (n = 5/group). **N** Flow cytometry histograms of dextran uptake in immature BMDCs (imBMDCs) treated with dexamethasone (Dex), NC-serum, or AA-serum, assessing endocytosis. **O** Quantification of relative endocytosis. Data are presented as means ± SEM, representing three experiments for flow cytometry analyses; ns, not significant; *P < 0.05, **P < 0.01 as indicated

To assess whether acupuncture directly affects T cell activity, T cells were stimulated with CD3/CD28 antibodies and treated with varying concentrations (10%, 1%, 0.1%) of serum from acupuncture-treated mice (AA-serum). CFSE dilution analysis showed that AA-serum had no significant effect on T cell proliferation (Fig. 3A, B), nor did it alter the percentage of activated CD25^+^CD69^+^ T cells, while cyclosporin A (CsA) markedly reduced activation (Fig. 3C, D). Furthermore, under Th2-polarizing conditions, AA-serum did not affect *Il4* or *Gata3* mRNA expression, indicating no direct effect on Th2 differentiation (Fig. 3E, F).

In contrast, AA-serum significantly inhibited LPS-stimulated BMDC maturation, as evidenced by the dose-dependent reduction in MHC II and CD86 expression, with the strongest effect at 10% AA-serum (Fig. 3G–J). Additionally, AA-serum suppressed the secretion of pro-inflammatory cytokines, including IL-1β, TNF-α, and IL-6, compared to NC-serum (Fig. 3K–M). Increased FITC-dextran uptake further suggested that AA-serum promoted immature BMDC phenotype (Fig. 3N, O). These results suggest that acupuncture does not directly target T cells but exerts immunomodulatory effects on DCs, which may contribute to its anti-inflammatory effects.

### Adoptive transfer of acupuncture-treated lung DCs alleviates HDM-induced allergic airway inflammation

**Fig. 4:**
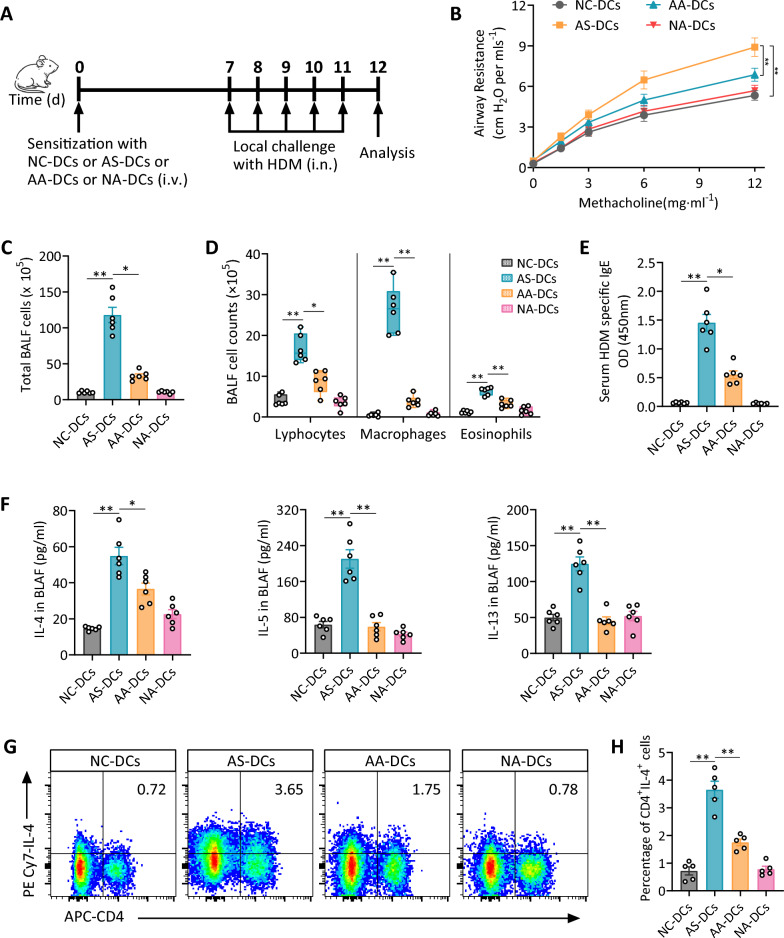
Acupuncture-treated lung DCs alleviates HDM-induced allergic airway inflammation. **A** Mice were sensitization with NC-DCs, AS-DCs, AA-DCs, or NA-DCs by intravenous (i.v.) injection, followed by five consecutive intranasal (i.n.) administrations of HDM for challenge. **B** Airway hyperresponsiveness to methacholine at incremental doses was analyzed 24 h after the final challenge. **C**, **D** Total and differential cell counts (lymphocytes, macrophages, and eosinophils) in BALF. **E**, **F** Serum HDM-specific IgE Levels (E) and Th2 cytokine concentrations (IL-4, IL-5, and IL-13) (F) in BALF were examined using ELISA assays. **G** Flow cytometry plots illustrating the percentage of CD4^+^IL-4^+^ Th2 cells in lung tissues, and **H** quantification of Th2 cells. Data are presented as means ± SEM; n = 5–6 mice/group; *P < 0.05, **P < 0.01 as indicated

To further investigate the immunomodulatory effects of acupuncture on lung DCs, an adoptive transfer experiment was conducted. Lung DCs from each experimental group were injected into normal mice followed by HDM challenge to induce asthma (Fig. 4A). Mice receiving AS-DCs exhibited increased airway resistance, whereas those receiving AA-DCs showed significantly lower resistance, particularly at higher MCh doses (Fig. 4B). The AA-DC group also had a marked reduction in total BALF cell counts, including lymphocytes, macrophages, and eosinophils compared to the AS-DC group (Fig. 4C, D). Serum levels of HDM-specific IgE were also significantly lower in the AA-DC group approaching levels observed in the NC-DC group (Fig. 4E).

Additionally, Th2 cytokine levels (IL-4, IL-5, and IL-13) in BALF were significantly reduced in AA-DC recipients compared to AS-DC recipients, further indicating attenuation of Th2-driven inflammation (Fig. 4F). Flow cytometry revealed a lower percentage of CD4^+^IL-4^+^ Th2 cells in AA-DC recipients, confirming the suppression of Th2-mediated inflammation (Fig. 4G, H). In contrast, the NA-DC group exhibited minimal Th2 inflammation, resembling the NC-DC group, suggesting acupuncture primarily exerts its effects in asthma conditions.

Collectively, these findings indicate that adoptive transfer of lung DCs from acupuncture-treated asthmatic mice effectively reduces Th2 airway inflammation, suggesting acupuncture modulates DC immune function to alleviate allergic inflammation.

### Acupuncture selectively influences CD11b⁺ DC subpopulations and their immunological activity in asthmatic mice

**Fig. 5:**
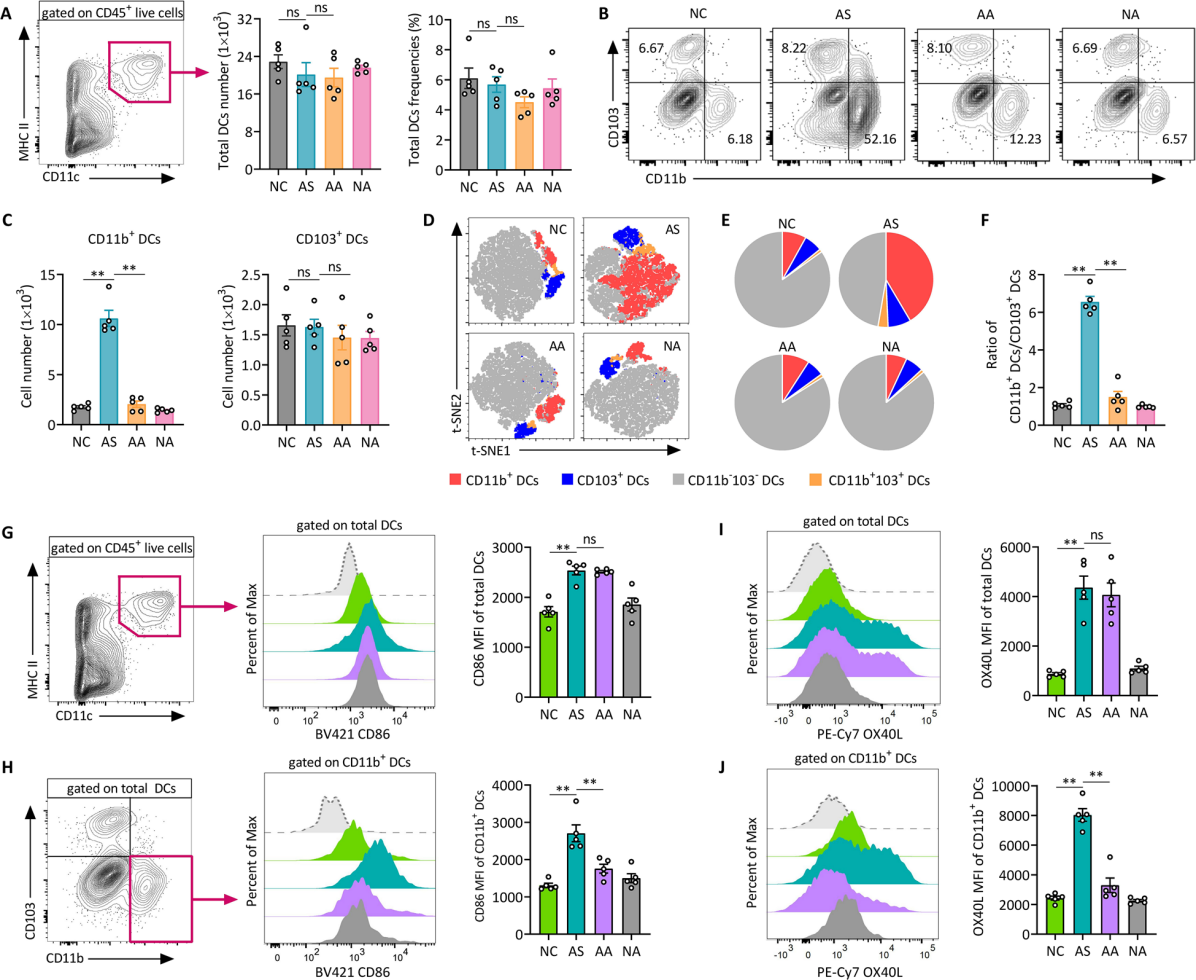
Acupuncture treatment reduces both the population and immune activation of lung CD11b⁺ DCs in allergic asthma. **A** Gating strategy for identifying total DCs from CD45^+^ live cells, along with the analysis of total DC numbers and frequencies. **B** Representative flow cytometry plots showing lung CD11b^+^CD103^−^ and CD11b^−^CD103^+^ DC subsets (gated on live CD45^+^CD11c^+^MHC II^+^ cells), and **C** quantitative analysis of CD11b⁺ and CD103⁺ DC subsets. **D** t-distributed stochastic neighbor embedding (t-SNE) plot illustrating the distribution of total lung DCs based on flow cytometry analysis. Cells are colored according to the four identified clusters. **E** Pie chart representation of DC subset composition in lungs. **F** Ratio of CD11b^+^ DCs to CD103^+^ DCs in the lung tissues. **G** Flow cytometry analysis of CD86 expression on total DCs, with representative histograms and MFI quantification. **H** Flow cytometry analysis of CD86 expression specifically on CD11b^+^ DCs, including representative histograms and MFI quantification. **I**,** J** Flow cytometry overlay histogram and MFI analysis of OX40L expression on total DCs (I) and CD11b^+^ DCs (J). Data are presented as means ± SEM; n = 5 mice/group; ns, not significant; *P < 0.05, **P < 0.01 as indicated

Flow cytometry analysis showed no changes in total lung DC counts across groups (Fig. 5A)**,** gating strategy shown in Fig. S3 A, but revealed a notable increase in CD11b⁺CD103⁻ DCs (CD11b^+^ DCs) in the AS group, which was significantly reduced by acupuncture treatment, restoring levels closer to those of NC group (Fig. 5B, C). In contrast, CD11b⁻CD103⁺ DCs (CD103^+^ DCs) remained unchanged, suggesting acupuncture selectively modulates the CD11b⁺ DC subset. t-distributed stochastic neighbor embedding (t-SNE) analysis confirmed this distribution, demonstrating an increased CD11b⁺ DC population in asthma, which was attenuated following acupuncture (Fig. 5D, E). Additionally, the ratio of CD11b⁺ to CD103⁺ DCs, an indicator of inflammatory status, was significantly elevated in the AS group and reduced by acupuncture, further supporting the selective regulatory effect of acupuncture on CD11b⁺ DCs (Fig. 5F).

To evaluate lung DC immune activity, the expression levels of the costimulatory molecules CD86 and OX40L were examined. The AS group exhibited a significant increase in CD86 expression in both total DCs and specifically within the CD11b⁺ DC subset, indicating enhanced activation of CD11b⁺ DCs in asthma. Acupuncture treatment significantly reduced CD86 expression in CD11b⁺ DCs, while total DCs levels remained unchanged (Fig. 5G, H). Similarly, OX40L levels were elevated in CD11b⁺ DCs in the AS group and significantly reduced following acupuncture treatment (Fig. 5I, J). Notably, neither CD86 nor OX40L expression was altered in the CD103⁺ DC subset (Fig. S4), further reinforcing that acupuncture specifically targets the CD11b⁺ DC population and its immunostimulatory activity, thereby mitigating airway inflammation in asthma.

### Acupuncture modulates CD11b^+^ DC migration and reduces pro-inflammatory chemokine and cytokine expression

**Fig. 6:**
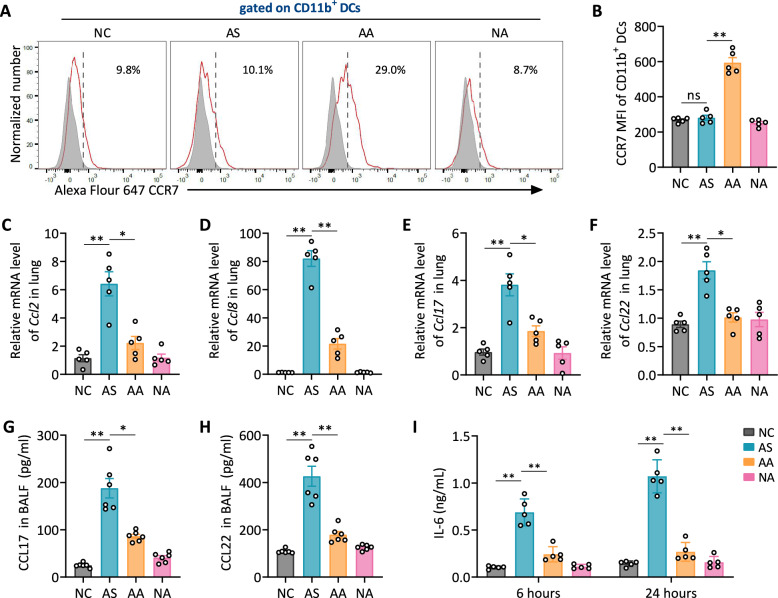
Acupuncture modulates lung CD11b^+^ DC migration and suppresses pro-inflammatory chemokine and cytokine expression. **A** Representative histograms depicting CCR7 expression on lung CD11b⁺ DCs. **B** MFI quantification of CCR7 expression in lung CD11b⁺ DCs. **C**–**F** Relative mRNA levels of *Ccl2*, *Ccl8*, *Ccl17*, and *Ccl22* in lung tissues, measured by RT-qPCR. **G**–**H** ELISA quantification of CCL17 and CCL22 in BALF. **I** IL-6 production by lung DCs following HDM restimulation in vitro at 6 h and 24 h. Data are presented as means ± SEM; n = 5–6 mice/group; ns, not significant; *P < 0.05, **P < 0.01 as indicated

CCR7 expression, crucial for CD11b⁺ DC migration to lymph nodes, was significantly upregulated in the AA group compared to the AS and NC groups, indicating enhanced migratory potential of lung CD11b⁺ DC following acupuncture treatment (Fig. 6A, B). Notably, lung CD11b^+^ DCs from the AA group exhibited reduced MHC II and costimulatory molecules, indicating that increased CCR7 expression facilitate their migration to lymph nodes, potentially contributing to allergen tolerance and immune homeostasis. Conversely, the AS group exhibited significantly increased mRNA levels of CCR2 ligands CCL2 and CCL8, which drive CD11b⁺ DC recruitment during inflammation. Acupuncture significantly decreased the expression of these chemokines, indicating inhibition of CCR2-mediated CD11b^+^ DC accumulation in asthma (Fig. 6C, D).

Additionally, CD11b^+^ DCs are known to secrete Th2-attracting chemokines such as CCL17 and CCL22, which promote Th2 cell recruitment during allergic inflammation. The AS group exhibited higher mRNA expression of *Ccl17* and *Ccl22* (Fig. 6E, F)**,** along with elevated protein levels of these chemokines in BALF (Fig. 6G, H). Acupuncture effectively reduced both the mRNA and protein levels of these chemokines, indicating diminished Th2 cell recruitment and an overall alleviation of airway inflammation. Furthermore, lung DCs from the AS group exhibited elevated IL-6 levels at both 6 and 24 h post-HDM restimulation, which were markedly reduced by acupuncture treatment in the AA group (Fig. 6I).

### Acupuncture attenuates ILC2 accumulation and suppresses epithelial-derived alarmins in asthmatic mice

**Fig. 7:**
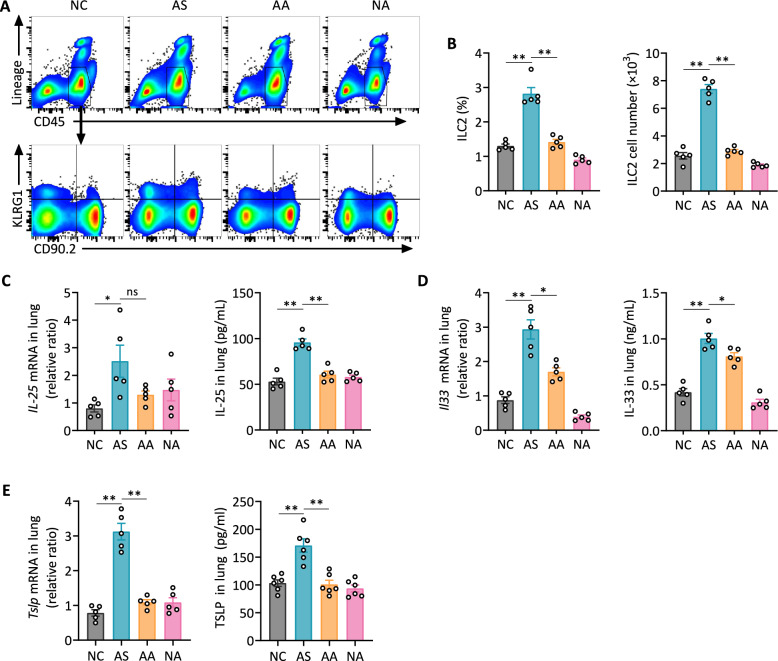
Acupuncture reduces ILC2 accumulation and suppresses epithelial-derived alarmins in the lung. **A** Representative flow cytometry plots identifying ILC2s in the lung, gated as Lineage⁻CD45⁺KLRG1⁺CD90.2⁺ cells.** B** Quantification of ILC2 percentage and absolute cell number in lung tissues. **C**–**E** Relative mRNA expression levels and protein concentrations of epithelial-derived alarmins IL-25 (C), IL-33 (D) and TSLP (**E**) in the lung. Data are presented as means ± SEM; n = 5 mice/group; *P < 0.05, **P < 0.01 as indicated

ILC2s drive eosinophilic airway inflammation by releasing Th2 cytokines such as IL-5 and IL-13, while also activating CD11b⁺ DCs to enhance memory Th2 responses. This study assessed whether acupuncture modulates ILC2 accumulation in allergic asthma. Flow cytometry identified ILC2s as lineage⁻CD45⁺CD90.2⁺KLRG1⁺ viable cells (Fig. S3B) and showed a significant HDM-induced increase in lung ILC2s in asthmatic mice, which was markedly reduced by acupuncture treatment (Fig. 7A, B).

Moreover, acupuncture suppressed lung IL-33 and IL-25 expression, both of which are critical alarmins involved in ILC2 activation, at both mRNA and protein levels (Fig. 7C, D), though *Il25* mRNA levels did not show a statistically significant difference between AS and AA groups. TSLP, which amplifies the effects of IL-33 and IL-25 on ILC2s, was also downregulated by acupuncture (Fig. 7E). These results indicate that acupuncture inhibits ILC2 activity, potentially reducing CD11b⁺ DC immune activation and alleviating Th2 airway inflammation in asthma.

## Discussion

Traditional Chinese medicine (TCM) has long used acupuncture to manage respiratory diseases, including asthma. Acupuncture, by stimulating specific acupoints, aims to restore *Qi* flow and balance bodily energy, enhancing respiratory function. Both the World Health Organization and the National Institutes of Health recognize acupuncture as an effective treatment for asthma [15, 16]. Recent meta-analyses support this, reporting that acupuncture improves quality of life, lung function (FEV1%), symptom scores, and Asthma Control Test scores, while reducing exacerbation frequency [17]. Our analysis of numerous clinical controlled trials [8], along with our previous clinical and experimental studies [9, 12], consistently demonstrated that the acupoints GV14, BL12, and BL13 produce significant therapeutic effects in asthma. Thus, these acupoints were selected for this study.

Our results show that acupuncture at GV14, BL12, and BL13 significantly improves lung function in an HDM-induced asthma model by reducing R_L_, enhancing C_dyn_, and decreasing immune cell infiltration in BALF, including lymphocytes, macrophages, and eosinophils. Histological analysis reveals reduced airway inflammation and mucus hypersecretion. Acupuncture also mitigates Th2 airway inflammation by lowering Th2 cytokine levels and reducing the proportion of Th2 cells in lungs and MLN cultures. Consistent with prior findings, it decreases serum IgE, activated T cells, eosinophils, and Th17 cells [9, 10, 12]. Together, our findings underscore the therapeutic potential of acupuncture at GV14, BL12, and BL13 in asthma treatment, particularly by modulating immune responses and alleviating Th2-driven airway inflammation.

To assess potential non-specific effects of acupuncture on physiology and immunity, we included a non-asthmatic acupuncture (NA) group in our study. Compared to normal controls (NC), acupuncture in healthy mice did not significantly alter airway resistance, immune cell infiltration, or cytokine levels. Additionally, histological examination revealed no evidence of tissue damage or inflammation, indicating that acupuncture does not disrupt immune homeostasis under non-asthmatic conditions. This contrasting response highlights both the specific regulatory mechanisms of acupuncture in asthma and its inherent safety in a balanced physiological state.

Acupuncture serum, collected from treated animals, contains bioactive factors and signaling molecules influenced by acupuncture [18], and serves as a model to study its systemic effects [11, 19, 20]. In this study, serum from the AA group showed no direct effect on T cell proliferation or activation in vitro. However, it inhibited BMDC maturation and reduced pro-inflammatory cytokine production upon LPS stimulation, suggesting an indirect modulation of T cell responses through DC regulation. Adoptive transfer of lung DCs from acupuncture-treated mice into wild-type recipients further confirmed this role. After antigen challenge, these DCs led to a reduction in BALF inflammatory cells, type 2 cytokines, HDM-specific IgE, and mucus secretion, demonstrating that acupuncture suppresses Th2 airway inflammation by modulating lung DC function.

DCs originate from hematopoietic stem cells in the bone marrow and differentiate into distinct subtypes, including conventional DCs (cDCs) and plasmacytoid DCs (pDCs), which exhibit differences in both location and immunological function [21]. cDCs, particularly CD11b^+^ DCs in the lung submucosa, play a pivotal role in T cell priming and Th2 polarization by expressing Th2-promoting molecules like OX40L and T1/ST2, which are not typically expressed by CD103^+^ DCs or pDCs [4, 22–24]. CD11b^+^ DCs also express dectin-1, which recognizes HDM antigens and drives allergic airway inflammation by modulating chemokine expression [25]. This study demonstrates that acupuncture significantly reduces CD11b^+^ DC populations in the lungs of asthma models without altering CD103^+^ DCs, suggesting that acupuncture modulates CD11b^+^ DC differentiation to mitigate asthma progression. Additionally, acupuncture downregulates co-stimulatory molecules such as CD86 and OX40L on CD11b^+^ DCs, limiting their capacity to activate Th2 cells.

CCR7 expression on CD11b^+^ DCs is critical for their migration to lymph nodes via CCL19/CCL21 gradients, which are essential for antigen presentation and adaptive immune responses [26, 27]. Our results show that acupuncture upregulated CCR7 expression on CD11b^+^ DCs while simultaneously reducing their immunostimulatory activity, which may enhance immune tolerance and reducing local Th2 inflammation. Conversely, CCR2 facilitates CD11b^+^ DC recruitment to inflamed lungs through interactions with CCL2 and CCL8, both of which are highly expressed in asthma [28, 29]. Our study found that acupuncture downregulated CCL2 and CCL8 expression, likely reducing CCR2-mediated CD11b^+^ DC accumulation in inflamed airways by modulating the CCL2/CCR2 and CCL8/CCR2 pathways.

CD11b^+^ DCs are primary sources of type 2 chemokines CCL17 and CCL22, which recruit Th2 cells to the airways in allergic asthma, thus contributing to Th2 inflammation [6]. Acupuncture lowered CCL17 and CCL22 expression at both mRNA and protein levels, potentially limiting Th2 cell migration to the airways and alleviating inflammation. Additionally, CD11b^+^ DCs produce IL-6, which promotes Th2 and Th17 cell differentiation and activation [14, 30]. Our study showed that acupuncture inhibits IL-6 secretion upon in vitro antigen restimulation of lung DCs, suggesting its role in suppressing pro-inflammatory cytokine production from CD11b^+^ DCs and alleviating the Th2 response in asthma.

ILC2s produce IL-5, IL-9, and IL-13 in response to epithelial-derived signals (e.g., IL-25, IL-33, and TSLP) [31]. In asthma, ILC2s play a pivotal role in initiating and amplifying airway inflammation [32, 33]. ILC2s stimulate lung CD11b^+^ DCs to produce CCL17, promoting Th2 memory cell recruitment and exacerbating inflammation [6]. Our study found that acupuncture reduced the number of ILC2s in asthmatic lungs and suppressed their activation cytokines, including IL-33, IL-25, and TSLP. Given that ILC2s promote the activation of CD11b^+^ DCs, the inhibitory effects of acupuncture on ILC2s may indirectly modulate the immune activity of CD11b^+^ DCs, leading to a further reduction in Th2-mediated airway inflammation. These findings suggest that the ILC2–DC interaction plays a central role in acupuncture’s therapeutic effects on asthma.

TSLP, IL-33, and IL-25 are key epithelial-derived alarmins that drive allergic inflammation in response to allergen exposure [34–36]. Our findings suggest that acupuncture not only attenuates immune cell activation but may also restore epithelial homeostasis by reducing alarmin production. One potential mechanism is that by suppressing the activation of ILC2s and DCs, acupuncture disrupts the feedback loop that amplifies epithelial cytokine release. Alternatively, acupuncture may exert direct effects on epithelial cells via neuroimmune pathways, wherein sensory nerve stimulation transmits signals to the central nervous system, leading to vagal activation and neurotransmitter release [37, 38]. These neurochemical signals not only regulate immune cell function but may also enhance epithelial barrier integrity and suppress alarmin production.

This proposed mechanism aligns with the anatomical positioning of the acupuncture points used in this study (GV14, BL12, BL13), which are located near the cervical sympathetic trunk and the T1-T4 sympathetic chain. This spatial relationship suggests that acupuncture may exert its effects through sympathetic pathways, facilitating both systemic immune modulation via the hypothalamic–pituitary–adrenal (HPA) axis [39] and localized regulation of pulmonary DCs and ILC2s through lung-specific sympathetic networks. Such multi-level neuroimmune modulation may underlie acupuncture’s concurrent alleviation of airway inflammation and epithelial dysfunction. However, the precise mechanisms governing acupuncture-mediated epithelial-neuro-immune crosstalk, particularly the specific contributions of distinct neural subtypes to its anti-asthmatic effects, require further investigation.

## Conclusion

In summary, this study demonstrates that acupuncture reduces lung CD11b^+^ DC populations and activation, thereby inhibiting Th2 cell recruitment and activation. This effect likely occurs through the downregulation of epithelial-derived alarmins, including IL-33, IL-25, and TSLP, which diminishes ILC2 activation and consequently the CD11b^+^ DC-mediated inflammatory response. These findings highlight the multi-target effects of acupuncture in modulating airway epithelial cells, ILC2s, DCs, and Th2 cells in asthma. Future studies on acupuncture’s neural-immune interactions could deepen our understanding of its effects and advance acupuncture-based therapeutic targets and strategies for asthma.

## Supplementary Information

Supplementary Material 1

Supplementary Material 2

## Data Availability

The datasets included in the current study are available from the corresponding author upon reasonable request.

## Abbreviations

AHR: Airway hyperresponsiveness
BALF: Bronchoalveolar lavage fluid
BMDCs: Bone marrow-derived dendritic cells
C_dyn_: Lung dynamic compliance
CSFE: Carboxyfluorescein diacetate succinimidyl ester
DCs: Dendritic cells
HDM: House dust mite
ILC2 s: Type 2 innate lymphoid cells
MCh: Methacholine
MLN: Mediastinal lymph node
PAS: Periodic acid schiff
R_L_: Lung resistance
TSLP: Thymic stromal lymphopoietin
