# Correction: Modulation of lung CD11b^+^ dendritic cells by acupuncture alleviates Th2 airway inflammation in allergic asthma

**DOI:** 10.1186/s13020-025-01140-y

**Published:** 2025-06-25

**Authors:** Mi Cheng, Pan-Pan Shang, Dan-Dan Wei, Jie Long, Xue Zhang, Quan-Long Wu, Gabriel Shimizu Bassi, Yu Wang, Yan-Jiao Chen, Lei-Miao Yin, Yong-Qing Yang, Yu-Dong Xu

**Affiliations:** 1 Shanghai Research Institute of Acupuncture and Meridian, Yueyang Hospital of Integrated Traditional Chinese and Western Medicine, Shanghai University of Traditional Chinese Medicine, Shanghai, China; 2 School of Rehabilitation Science, Shanghai University of Traditional Chinese Medicine, Shanghai, China

**Correction: Chinese Medicine (2025) 20:67** 10.1186/s13020-025-01119-9

Following publication of the original article [1], the authors identified an error in the layout of Fig. 5D, the actual t-SNE plot that should display the distribution of Lung DCs is missing and appears blank.

The correct Fig. 5 has been provided in this Correction.

The incorrect Fig. 5 is:

**Fig. 5:**
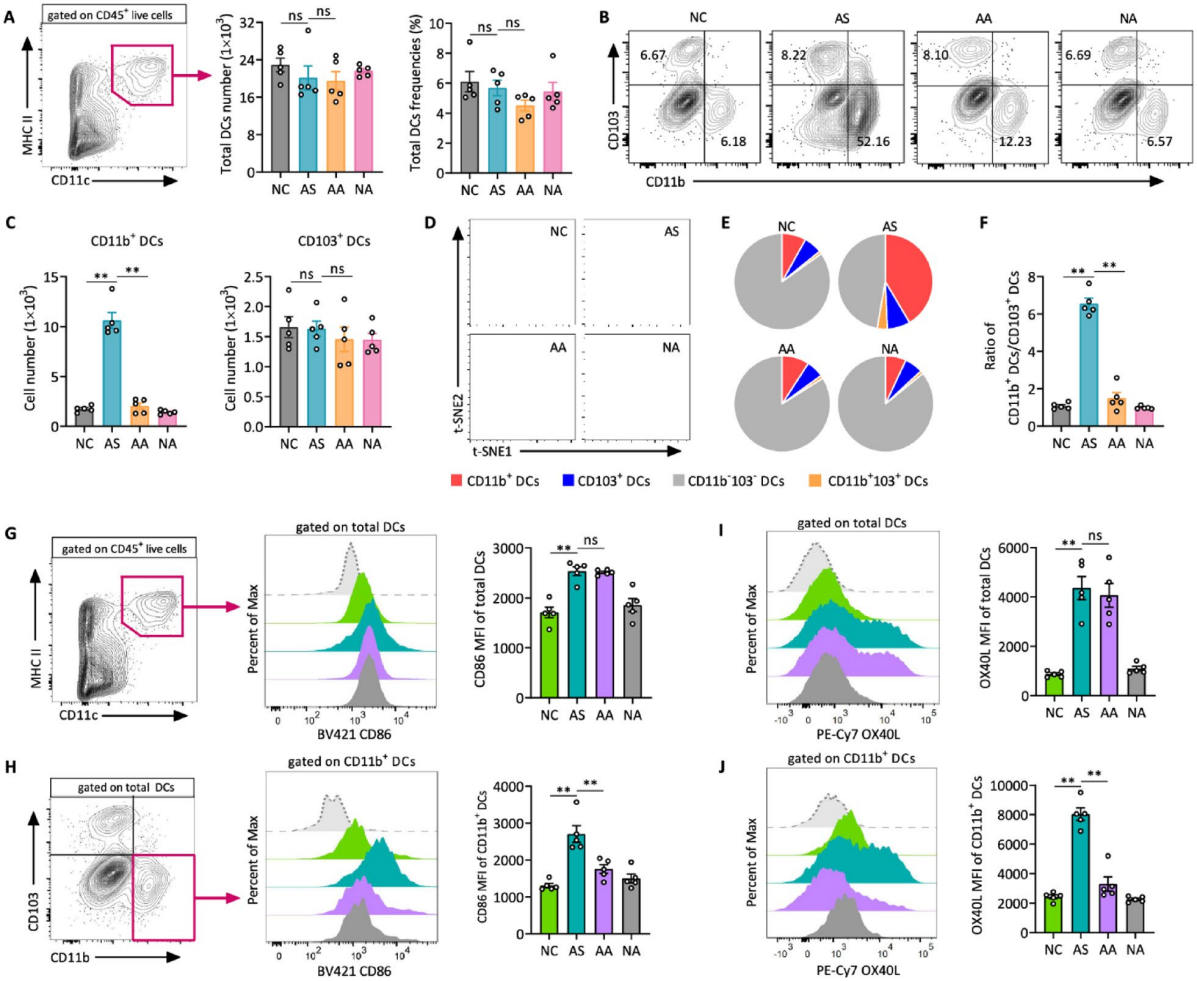
Acupuncture treatment reduces both the population and immune activation of lung CD11b⁺ DCs in allergic asthma. **A** Gating strategy for identifying total DCs from CD45^+^ live cells, along with the analysis of total DC numbers and frequencies. **B** Representative flow cytometry plots showing lung CD11b^+^CD103^−^ and CD11b−CD103+ DC subsets (gated on live CD45^+^CD11c^+^MHC II^+^ cells), and **C** quantitative analysis of CD11b⁺ and CD103⁺ DC subsets. **D** t-distributed stochastic neighbor embedding (t-SNE) plot illustrating the distribution of total lung DCs based on flow cytometry analysis. Cells are colored according to the four identified clusters. **E** Pie chart representation of DC subset composition in lungs.** F** Ratio of CD11b^+^ DCs to CD103^+^ DCs in the lung tissues. **G** Flow cytometry analysis of CD86 expression on total DCs, with representative histograms and MFI quantification. **H** Flow cytometry analysis of CD86 expression specifically on CD11b^+^ DCs, including representative histograms and MFI quantification. **I**,** J** Flow cytometry overlay histogram and MFI analysis of OX40L expression on total DCs (I) and CD11b^+^ DCs (J). Data are presented as means ± SEM; n = 5 mice/group; ns, not significant; *P < 0.05, **P < 0.01 as indicated

The correct Fig. 5 is:

**Fig. 5:**
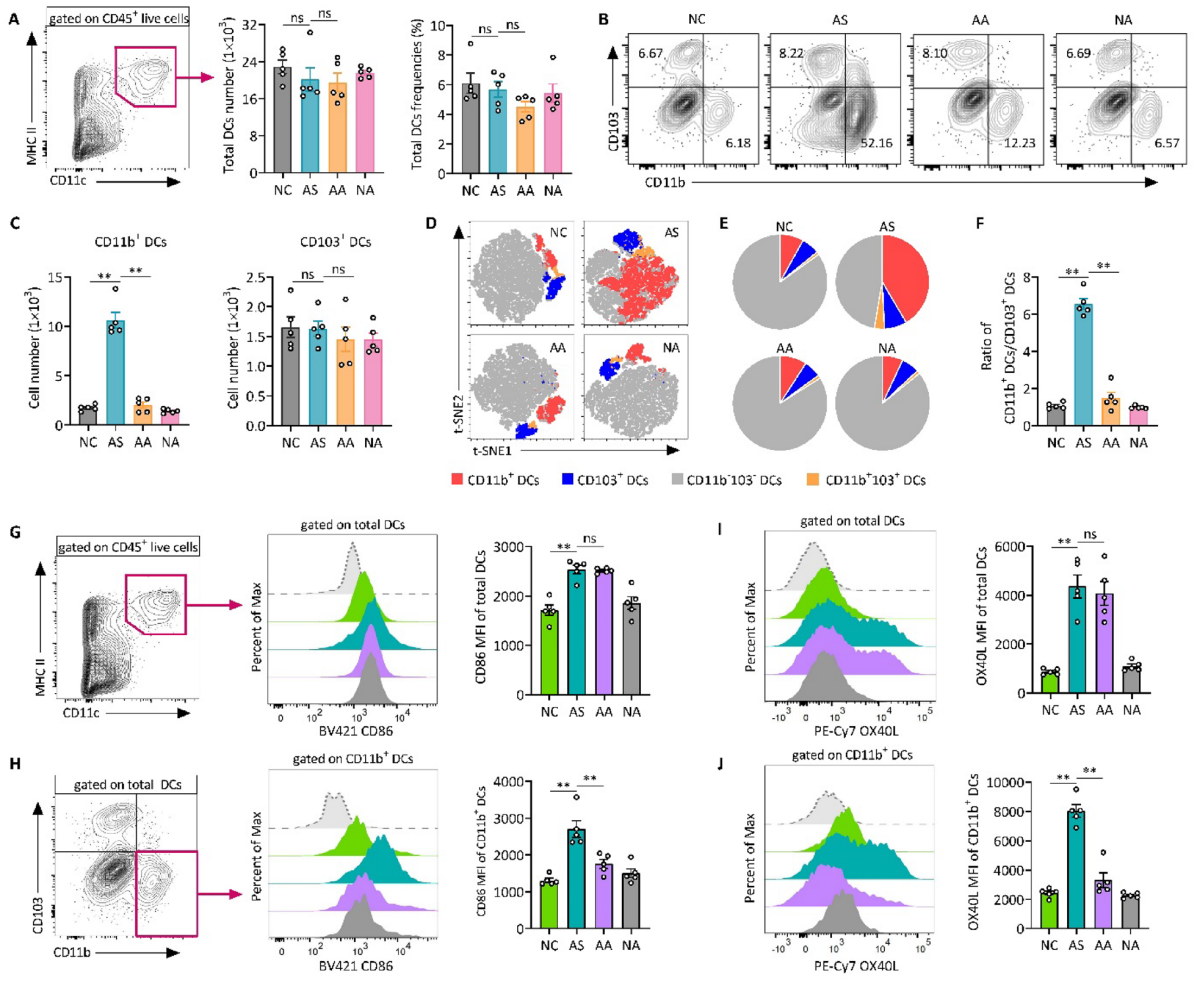
Acupuncture treatment reduces both the population and immune activation of lung CD11b⁺ DCs in allergic asthma. **A** Gating strategy for identifying total DCs from CD45^+^ live cells, along with the analysis of total DC numbers and frequencies. **B** Representative flow cytometry plots showing lung CD11b^+^CD103^−^ and CD11b^−^CD103^+^ DC subsets (gated on live CD45^+^CD11c^+^MHC II^+^ cells), and **C** quantitative analysis of CD11b⁺ and CD103⁺ DC subsets. **D** t-distributed stochastic neighbor embedding (t-SNE) plot illustrating the distribution of total lung DCs based on flow cytometry analysis. Cells are colored according to the four identified clusters. **E** Pie chart representation of DC subset composition in lungs. F Ratio of CD11b^+^ DCs to CD103^+^ DCs in the lung tissues. **G** Flow cytometry analysis of CD86 expression on total DCs, with representative histograms and MFI quantification. **H** Flow cytometry analysis of CD86 expression specifically on CD11b^+^ DCs, including representative histograms and MFI quantification. **I**,** J** Flow cytometry overlay histogram and MFI analysis of OX40L expression on total DCs (**I**) and CD11b^+^ DCs (**J**). Data are presented as means ± SEM; n = 5 mice/group; ns, not significant; *P < 0.05, **P < 0.01 as indicated

The original article [1] has been corrected.

## References

[1] Cheng M, Shang PP, Wei DD, et al. Modulation of lung CD11b^+^ dendritic cells by acupuncture alleviates Th2 airway inflammation in allergic asthma. Chin Med. 2025;20:67. 10.1186/s13020-025-01119-9. 40405264 10.1186/s13020-025-01119-9 PMC12100888

