# Supplementary material for: Modulation of lung CD11b+ dendritic cells by acupuncture alleviates Th2 airway inflammation in allergic asthma
Source: Chin Med. 2025 May 22;20:67. doi: 10.1186/s13020-025-01119-9 (PMC12100888; doi:10.1186/s13020-025-01119-9)
Supplement: Supplementary file 1 — Supplementary Material 1 [file 13020_2025_1119_MOESM1_ESM.docx]

**Modulation** **of lung CD11b^+^ dendritic cells by acupuncture alleviates Th2 airway inflammation in allergic asthma**

Mi Cheng^1†^, Pan-Pan Shang^1†^, Dan-Dan Wei^1†^, Jie Long^1^, Xue Zhang^1^, Quan-Long Wu^1^, Gabriel Shimizu Bassi^1, 2^, Yu Wang^1^, Yan-Jiao Chen^1^, Lei-Miao Yin^1^, Yong-Qing Yang^1*^, Yu-Dong Xu^1*^

1. Shanghai Research Institute of Acupuncture and Meridian, Yueyang Hospital of Integrated Traditional Chinese and Western Medicine, Shanghai University of Traditional Chinese Medicine, Shanghai, China

2. School of Rehabilitation Science, Shanghai University of Traditional Chinese Medicine, Shanghai, China

**†** These authors contributed equally to this work.

***** Corresponding author: Yu-Dong Xu, [xuyudong@shutcm.edu.cn](mailto:xuyudong@shutcm.edu.cn); Yong-Qing Yang, [yyq@shutcm.edu.cn](mailto:yyq@shutcm.edu.cn)

**Supplementary Tables 1-3**

**Table S1. The detailed information of ELISA kits used in this study**

| **ELISA Assay Kit** | **Source** | **Identifier** | **Lower Limit of**  **Quantification** | **Upper Limit of**  **Quantification** |
| --- | --- | --- | --- | --- |
| Mouse IL-4 | Biolegend | 431104 | 2 pg/mL | 125 pg/mL |
| Mouse IL-5 | Biolegend | 431204 | 7.8 pg/mL | 500 pg/mL |
| Mouse IL-6 | Biolegend | 431304 | 7.8 pg/mL | 500 pg/mL |
| Mouse IL-1β | Biolegend | 432604 | 31.3 pg/mL | 2000 pg/mL |
| Mouse TNF-α | Biolegend | 430904 | 7.8 pg/mL | 500 pg/mL |
| Mouse TSLP | Biolegend | 434104 | 7.8 pg/mL | 500 pg/mL |
| Mouse IL-25 | Biolegend | 447104 | 12.5 pg/mL | 800 pg/mL |
| Mouse IL-13 | Invitrogen | 88-7137 | 4 pg/mL | 500 pg/mL |
| Mouse IL-33 | Invitrogen | 88-7333 | 25 pg/mL | 3000pg/mL |
| Mouse CCL17 | R&D Sysytem | DY529-05 | 31.2 pg/mL | 2000 pg/mL |
| Mouse CCL22 | PeproTeck | 900-K197 | 7.8 pg/mL | 1000 pg/mL |

**Table S2.** **Primer sequences for RT-qPCR in this study**

| **Gene** | **Primer sequence** | **Product size (bp)** |
| --- | --- | --- |
| *Il4* | Forward 5’ CATCGGCATTTTGAACGAGGT 3’ | 100 |
|  | Reverse 5’ CTCACTCTCTGTGGTGTTCTTC 3’ |  |
| *Il5* | Forward 5’ CTCTGTTGACAAGCAATGAGACG 3’ | 102 |
|  | Reverse 5’ TCTTCAGTATGTCTAGCCCCTG 3’ |  |
| *Il13* | Forward 5’ CACACAAGACCAGACTCCCCT 3’ | 145 |
|  | Reverse 5’ GCCATGCAATATCCTCTGGGT 3’ |  |
| *Gata3* | Forward 5’ ACCACCTATCCGCCCTATGT 3’ | 104 |
|  | Reverse 5’ TTGGGCCTCGACTTACATCC 3’ |  |
| *Muc5ac* | Forward 5’ CCACTTTCTCCTTCTCCACACC 3’ | 119 |
|  | Reverse 5’ GGTTGTCGATGCAGCCTTGCTT 3’ |  |
| *Muc5b* | Forward 5’ CTGAAGACCTGTCGGAACCCAA 3’ | 122 |
|  | Reverse 5’ GCCACACACTTCATCTGGTCCT 3’ |  |
| *Ccl2* | Forward 5’ CACTCACCTGCTGCTACTCA 3’ | 117 |
|  | Reverse 5’ GCTTGGTGACAAAAACTACAGC 3’ |  |
| *Ccl8* | Forward 5’ CTACGCAGTGCTTCTTTGCC 3’ | 85 |
|  | Reverse 5’ GGTGACTGGAGCCTTATCTGG 3’ |  |
| *Ccl22* | Forward 5’ ACCTCTGATGCAGGTCCCTAT 3’ | 86 |
|  | Reverse 5’ AAACGTGATGGCAGAGGGTG 3’ |  |
| *Ccl17* | Forward 5’ AGACCTTCACCTCAGCTTTTG 3’ | 144 |
|  | Reverse 5’ CTTTGAAGTAATCCAGGCAGC 3’ |  |
| *Tslp* | Forward 5’ TCTCAGGAGCCTCTTCATCCT 3’ | 153 |
|  | Reverse 5’ ATTTGCTCGAACTTAGCCCCT 3’ |  |
| *Il25* | Forward 5’ CCTTGGAGCTATGAGTTGGAC 3’ | 141 |
|  | Reverse 5’ GTGGTAAAGTGGGACGGAGTT 3’ |  |
| *Il33* | Forward 5’ TTCCAACTCCAAGATTTCCCC 3’ | 134 |
|  | Reverse 5’ CAGAACGGAGTCTCATGCAG 3’ |  |
| *Gapdh* | Forward 5’ TCCCAGCTTAGGTTCATCAGG 3’ | 87 |
|  | Reverse 5’ CCAAATCCGTTCACACCGAC 3’ |  |

**Table S3. The detailed information of the FACS antibodies used in this study**

| **Antibodies** | **Clone** | **Dilution** | **Source** | **Identifier** |
| --- | --- | --- | --- | --- |
| PE-Cyanine7 anti-mouse CD25 | PC61 | 1: 200 | BD Biosciences | 552880 |
| FITC anti-mouse I-A/I-E | 2G9 | 1: 250 | BD Biosciences | 553623 |
| BUV 395 anti-mouse CD11c | HL3 | 1: 200 | BD Biosciences | 564080 |
| Alexa Fluor 647 anti-mouse CCR7 | 4B12 | 1: 200 | BD Biosciences | 560766 |
| PE anti-mouse CD86 | GL1 | 1: 200 | BD Biosciences | 553692 |
| FITC anti-Mouse CD3 | 17A2 | 1: 400 | BD Biosciences | 561798 |
| PE-Cyanine7 anti-mouse IL-4 | 11B11 | 1: 200 | BD Biosciences | 560699 |
| PE anti-Mouse IL-17A | TC11-18H10 | 1: 200 | BD Biosciences | 559502 |
| APC anti-mouse CD4 | RM4-5 | 1: 200 | BD Biosciences | 553051 |
| APC anti-mouse CD69 | H1.2F3 | 1: 200 | BD Biosciences | 560689 |
| Brilliant Violet 421 anti-mouse CD86 | GL1 | 1: 200 | Biolegend | 105032 |
| PE-Cyanine7 anti-mouse OX40L | RM134L |  | 1: 200 | Biolegend |
| PE anti-mouse CD103 | 2E7 | 1: 200 | Biolegend | 121406 |
| PerCP/Cyanine5.5 anti-mouse CD11b | M1/70 | 1: 200 | Biolegend | 101228 |
| Brilliant Violet 510 anti-mouse CD45 | 30-F11 | 1: 200 | Biolegend | 103138 |
| Brilliant Violet 421 anti-mouse KLRG1 | 2F1 | 1: 200 | Biolegend | 138414 |
| PerCP/Cyanine5.5 anti-mouse CD45 | 30-F11 | 1: 200 | eBioscience | 45-0451-82 |
| APC anti-mouse 90.2 | 53-2.1 | 1: 200 | Invitrogen | 17-0902-81 |
| FITC-mouse hematopoietic lineage antibody cocktail | 17A2, RA3-6B2, M1/70, TER-119, RB6-8C5 | 20µL/Test | Invitrogen | 22-7770-72 |
| Fixable Viability Dye eFlour 780 | - | 1:500 | Invitrogen | 65-0865-14 |
