# Supplementary material for: Modulation of lung CD11b+ dendritic cells by acupuncture alleviates Th2 airway inflammation in allergic asthma
Source: Chin Med. 2025 May 22;20:67. doi: 10.1186/s13020-025-01119-9 (PMC12100888; doi:10.1186/s13020-025-01119-9)
Supplement: Supplementary file 2 — Supplementary Material 2 [file 13020_2025_1119_MOESM2_ESM.docx]

**Modulation** **of lung CD11b^+^ dendritic cells by acupuncture alleviates Th2 airway inflammation in allergic asthma**

Mi Cheng^1†^, Pan-Pan Shang^1†^, Dan-Dan Wei^1†^, Jie Long^1^, Xue Zhang^1^, Quan-Long Wu^1^, Gabriel Shimizu Bassi^1, 2^, Yu Wang^1^, Yan-Jiao Chen^1^, Lei-Miao Yin^1^, Yong-Qing Yang^1*^, Yu-Dong Xu^1*^

1. Shanghai Research Institute of Acupuncture and Meridian, Yueyang Hospital of Integrated Traditional Chinese and Western Medicine, Shanghai University of Traditional Chinese Medicine, Shanghai, China

2. School of Rehabilitation Science, Shanghai University of Traditional Chinese Medicine, Shanghai, China

**†** These authors contributed equally to this work.

***** Corresponding author: Yu-Dong Xu, [xuyudong@shutcm.edu.cn](mailto:xuyudong@shutcm.edu.cn); Yong-Qing Yang, [yyq@shutcm.edu.cn](mailto:yyq@shutcm.edu.cn)

**Supplementary Figures 1-4**

**Figure S1**


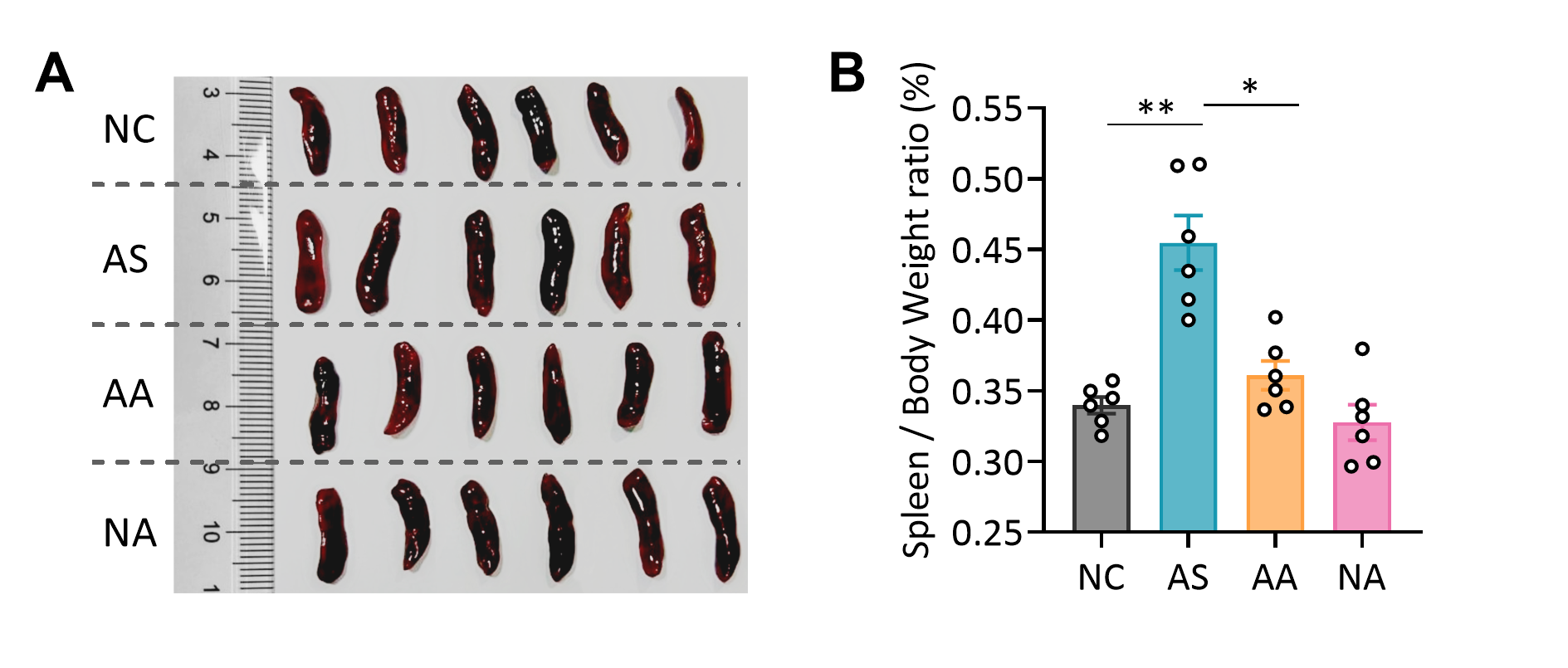


**Figure S1. Effect of acupuncture on spleen size and spleen-to-body weight ratio in an HDM-induced asthma model.**

*(A)* Representative images of spleens from each experimental group. Groups include NC (normal control), AS (asthma model), AA (asthma model treated with acupuncture), and NA (normal control treated with acupuncture).

*(B)* Quantification of the spleen-to-body weight ratio (%) across groups. Data are presented as mean ± SEM. Statistical significance was analyzed using one-way ANOVA (n = 6 mice/group), with **p < 0.01 and *p < 0.05. The AS group shows a significant increase in spleen-to-body weight ratio compared to the NC group. Acupuncture treatment (AA) notably reduces the spleen-to-body weight ratio relative to the AS group, suggesting a regulatory effect of acupuncture on spleen hypertrophy associated with asthma-induced inflammation.

**Figure S2**


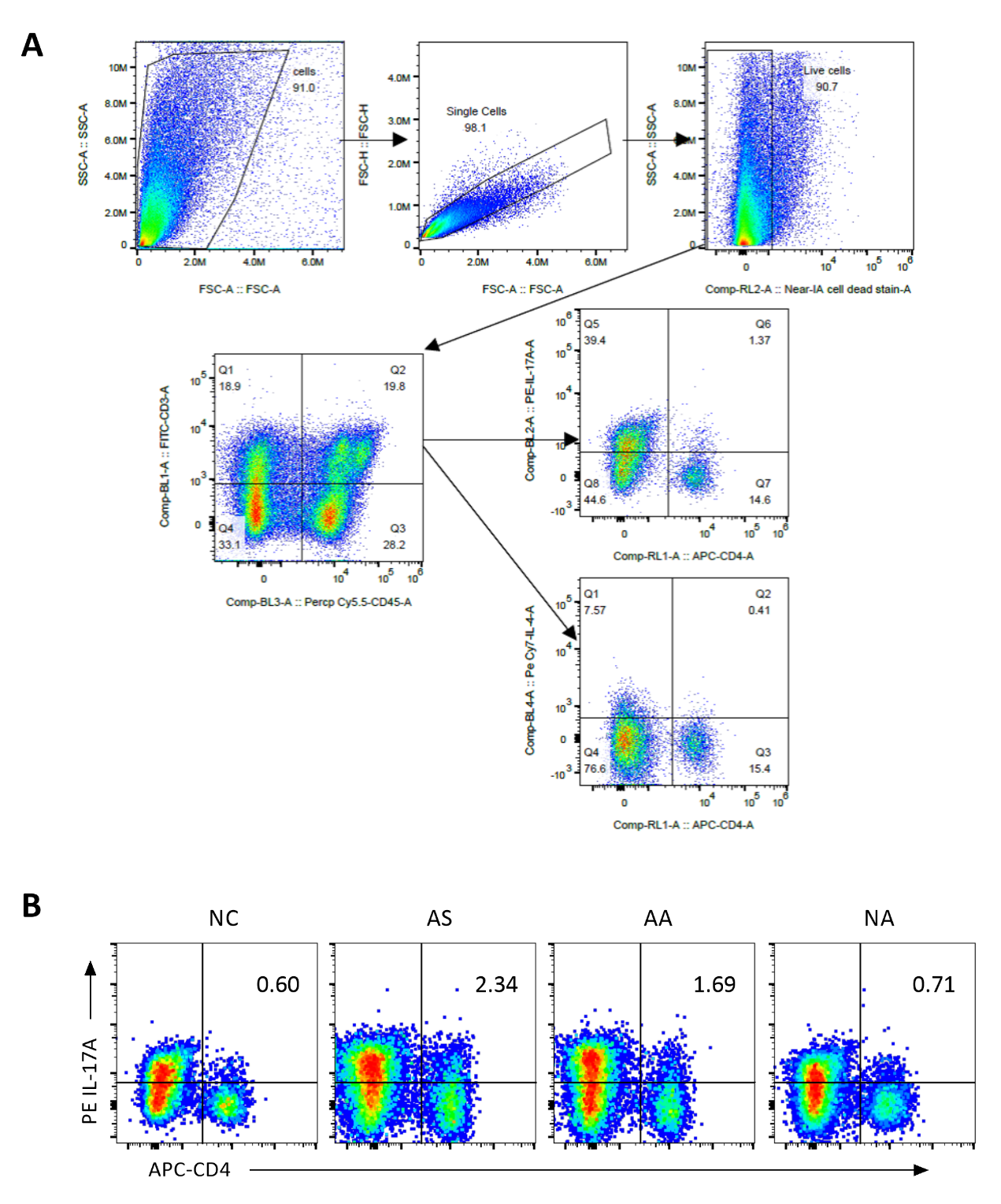


**Figure S2. Flow cytometry analysis of CD4⁺IL-17A⁺ T cells in lung tissue.**

*(A)* Flow cytometry gating scheme for Th2 and Th17 cells. T cells were identified as CD3^+^CD45^+^ cells and were gated out of singlet live cells. Th2 and Th17 cells were further discriminated according to their intracellular marker expression, by which CD4^+^IL-4^+^ cells were considered as Th2 cells, and CD4^+^IL-17A^+^ cells considered as Th2 cells.

*(B)* Representative dot plots showing the frequency of CD4⁺IL-17A⁺ T cells in each group. The asthma model (AS) group exhibits a higher percentage of CD4⁺IL-17A⁺ T cells compared to the control, while acupuncture treatment (AA) reduces the proportion of these cells, suggesting an anti-inflammatory effect of acupuncture on Th17-mediated airway inflammation.

**Figure S3**


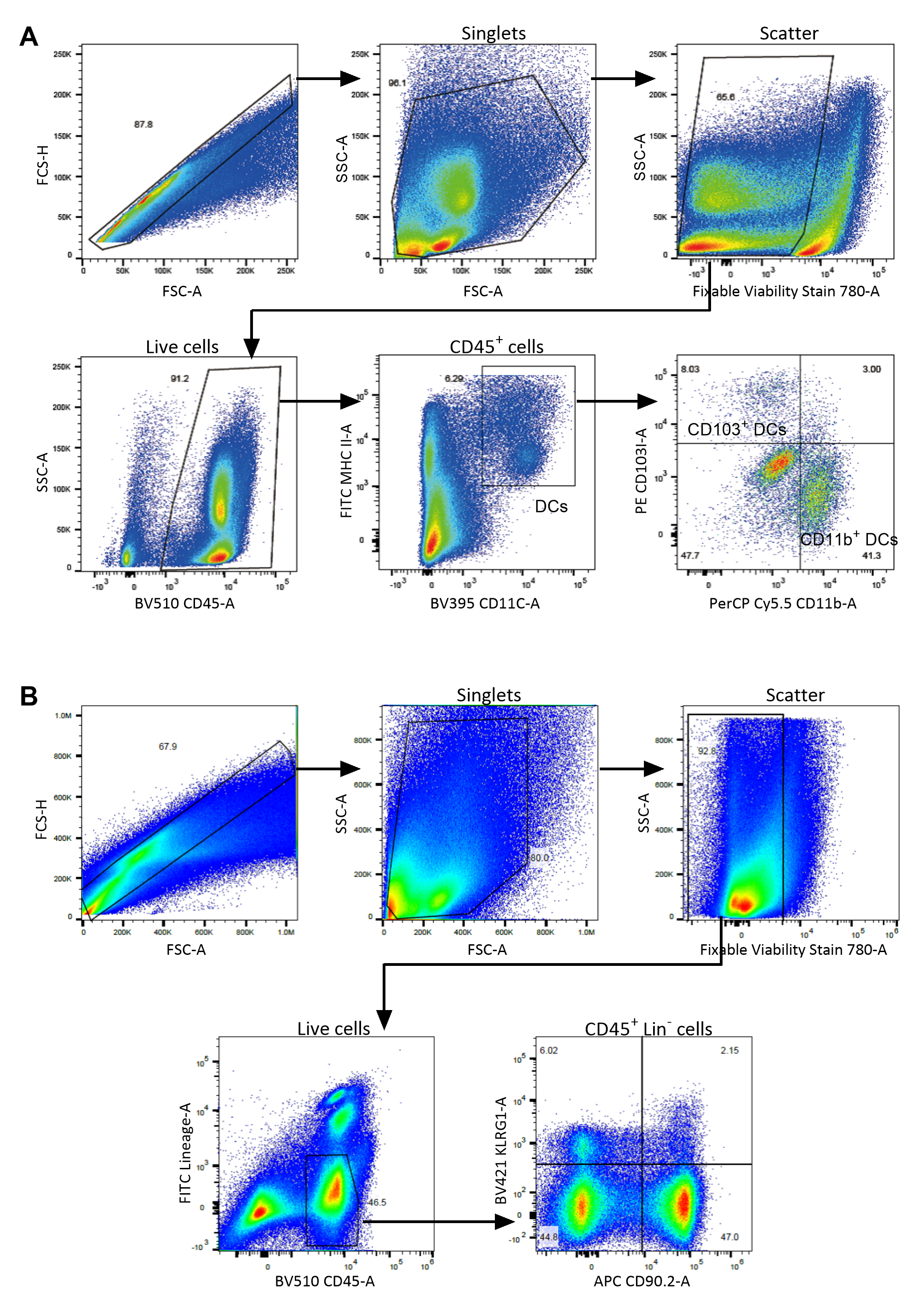


**Figure S3. Flow cytometry gating strategy for identifying dendritic cell (DC) subsets and type 2 innate lymphoid cells (ILC2s) in lung tissue.**
*(A)* Gating strategy for CD11b⁺ and CD103⁺ DC subsets. DCs were identified as CD11c^+^MHC II^+^ cells and were gated out of singlet live CD45^+^ cells. Among the DC population, CD11b and CD103 markers were used to distinguish CD11b⁺ DCs and CD103⁺ DCs subsets.

*(B)* Gating strategy for ILC2s. Lung cells were initially gated to remove doublets and dead cells, followed by gating on CD45⁺ to isolate immune cells. Lineage-negative (Lin⁻) cells were identified to exclude non-ILC populations. Within the CD45^+^Lin⁻ population, ILC2s were gated based on KLRG1 and CD90.2 expression.

**Figure S4**


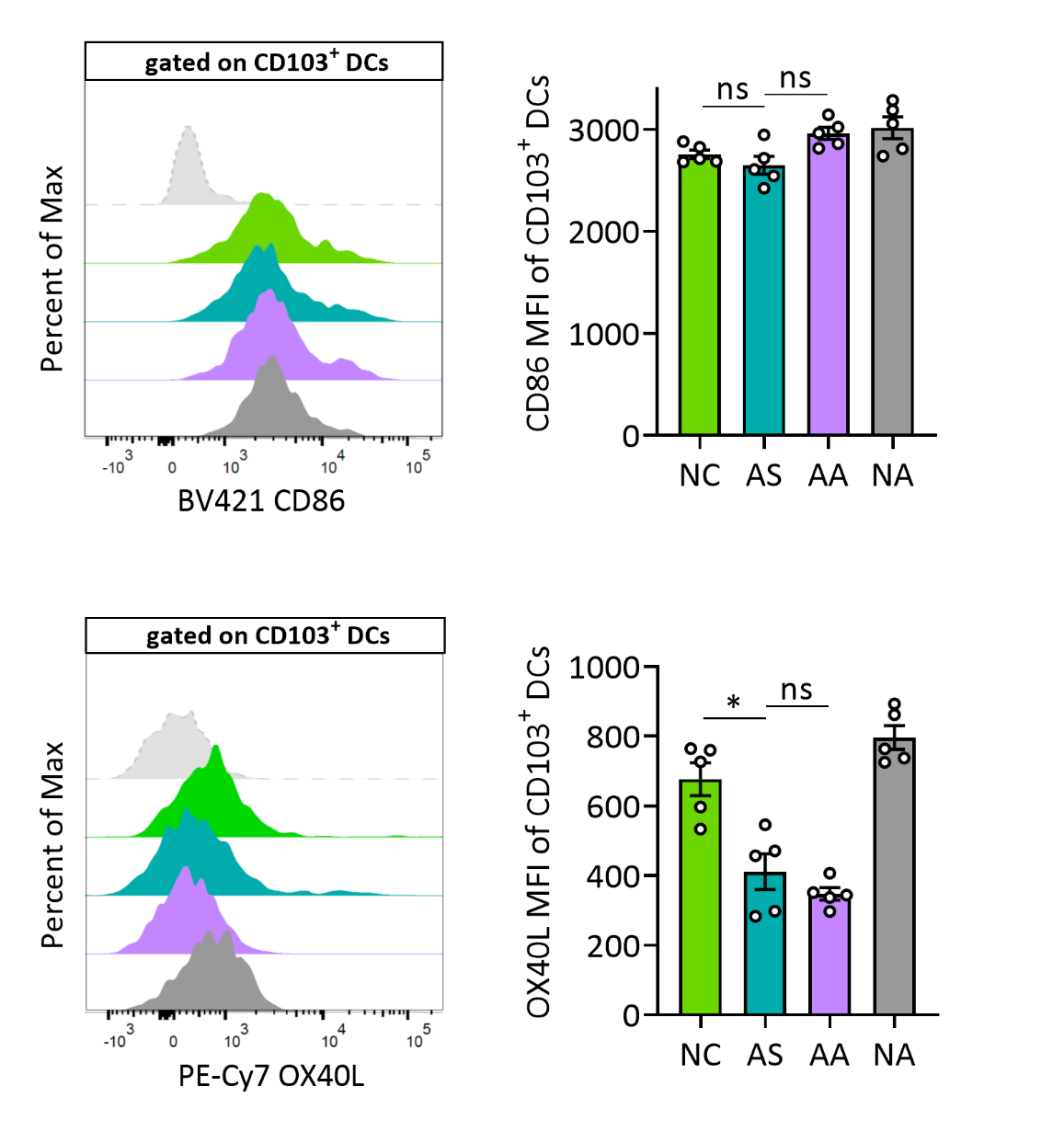


**Figure S4. Acupuncture does not affect CD86 and OX40L expression in the CD103^+^ DC subset.**

*(A)* Representative flow cytometry histograms showing CD86 expression in CD103^+^ DCs. The corresponding bar graph represents the mean fluorescence intensity (MFI) of CD86 in CD103^+^ DCs.

*(B)* Representative flow cytometry histograms showing OX40L expression in CD103^+^ DCs. The bar graph represents the MFI of OX40L in CD103^+^ DCs. OX40L expression was significantly reduced in the AS group compared to the NC group (*p < 0.05). However, acupuncture treatment did not significantly alter OX40L expression in the AA group compared to the AS group.

Data are presented as mean ± SEM, n = 5 mice/group. Statistical analysis was performed using one-way ANOVA followed by LSD or Games-Howell post-hoc test. ns, not significant.
